# A gene signature for post-infectious chronic fatigue syndrome

**DOI:** 10.1186/1755-8794-2-38

**Published:** 2009-06-25

**Authors:** John W Gow, Suzanne Hagan, Pawel Herzyk, Celia Cannon, Peter O Behan, Abhijit Chaudhuri

**Affiliations:** 1 Dept. of Biological and Biomedical Sciences, Glasgow Caledonian University, Glasgow, G4 0BA, UK; 2 The Sir Henry Wellcome Functional Genomics Facility, Faculty of Biomedical and Life Sciences, University of Glasgow, Glasgow, G12 8QQ, UK; 3 Glasgow Veterinary School, University of Glasgow, Glasgow, UK; 4 Essex Centre for Neurological Sciences, Oldchurch Hospital, Romford, RM7 0BE, UK

## Abstract

**Background:**

At present, there are no clinically reliable disease markers for chronic fatigue syndrome. DNA chip microarray technology provides a method for examining the differential expression of mRNA from a large number of genes. Our hypothesis was that a gene expression signature, generated by microarray assays, could help identify genes which are dysregulated in patients with post-infectious CFS and so help identify biomarkers for the condition.

**Methods:**

Human genome-wide Affymetrix GeneChip arrays (39,000 transcripts derived from 33,000 gene sequences) were used to compare the levels of gene expression in the peripheral blood mononuclear cells of male patients with post-infectious chronic fatigue (n = 8) and male healthy control subjects (n = 7).

**Results:**

Patients and healthy subjects differed significantly in the level of expression of 366 genes. Analysis of the differentially expressed genes indicated functional implications in immune modulation, oxidative stress and apoptosis. Prototype biomarkers were identified on the basis of differential levels of gene expression and possible biological significance

**Conclusion:**

Differential expression of key genes identified in this study offer an insight into the possible mechanism of chronic fatigue following infection. The representative biomarkers identified in this research appear promising as potential biomarkers for diagnosis and treatment.

## Background

Persistent fatigue after infection is recognised and forms part of a clinically-defined syndrome (chronic fatigue syndrome or CFS). CFS is a highly heterogeneous illness which is characterised by the presence of new-onset, persistent or relapsing fatigue of sufficient severity that interferes with normal activity. Patients also report impaired short-term memory and concentration, muscle pain and post-exertional malaise [1]. The clinical incidence of CFS in the population ranges from 0.23–2% and nearly 75% of patients are female [2,3]. The cause and pathogenesis of CFS are not understood and, to date, no standard laboratory test reliably distinguishes CFS patients from healthy subjects. As a result, persistence of otherwise medically-unexplained chronic fatigue often has been attributed to psychological factors.

In recent years, numerous microarray studies have been undertaken in order to distinguish patients with chronic fatigue from healthy controls. This subject has also been covered extensively in a special 2006 issue of the journal Pharmacogenomics. In this issue, several papers utilised numerous multivariate projection methods, bioinformatics, algorithms and computational analyses of microarray data, in order to provide better discrimination of subjects with unexplained chronic fatigue and CFS, from healthy controls [4-7].

Although chronic fatigue is well recognised after certain infections (for example, Lyme disease and Epstein-Barr virus), CFS is not commonly considered to be due to persistent infection. A preceding history of non-infection is frequently reported and recent studies indicate other factors may be responsible for the symptoms of this condition [8,9]. As a result, few studies have specifically addressed changes in gene expression in post-infectious subjects [10,11].

We hypothesised that patients with persistent fatigue developing after community-acquired infections are biologically different from healthy subjects and this would be reflected in a differential gene signature. Recent advances in genome sequencing and automated chip manufacture have made DNA chip or microarray technology readily available [12]. This technology allows simultaneous differential expression profiling from a large number of genes in tissue samples of CFS patients and controls. A previous study, using microarray technology encoding 1,764 genes and RNA from peripheral blood mononuclear cells (PBMC), demonstrated the utility of white blood cells for gene expression profiling on an illness without a known pathological lesion, such as CFS [13]. A more recent microarray study utilising 9,522 genes concluded that patients with CFS have reproducible alterations in gene regulation [14]. In addition, a study of exercise-responsive genes using a 3,800 oligonucleotide microarray showed significant differences in membrane ion transport genes in women with CFS, as compared to control subjects [15].

In our study, we aimed to obtain a complete "gene signature" for non-psychiatric patients with post-infectious persistent chronic fatigue. We excluded female subjects in order to avoid confounding factors from the monthly reproductive cycle. By using an Affymetrix GeneChip Human Genome U133 double-chip set that contains nearly 45,000 probe sets, representing 39,000 transcripts derived from more than 33,000 human gene sequences, most of the known human genome was encompassed in this work.

## Methods

### Study subjects

All patients underwent full medical and neurological evaluation clinically and had appropriate investigations to exclude alternative explanations for their symptoms. All patients with CFS fulfilled the international research criteria for diagnosis [1]. CFS patients and healthy subjects were closely matched for age, ethnicity and for place of residence in a common geographic area (central Scotland). Eight male patients aged between 18 and 54 years (median 36 years) with well-characterised, post-infectious CFS (median duration 4.5 years), after a documented history of viral or bacterial infections, were still independent, not clinically anxious or depressed and not taking regular medication were selected. Seven asymptomatic and physically active males aged between 22 and 58 years (median 34 years), with no recent history of infection, were used as healthy controls. Female participants at this stage were deliberately excluded to avoid confounding factors from their reproductive cycles and contraception. Informed consent and local ethical approval were obtained.

### Specimens

Male patients with post-infectious CFS and normal healthy controls underwent venous blood sample collection (prior to 11 am) in 2 × 5 ml EDTA tubes. PBMC were isolated immediately and stored within 2 hours of sampling. EDTA-treated whole blood was diluted 1:1 with phosphate buffered saline (PBS). Two volumes of blood were overlaid onto one volume Histopaque-1077 (Sigma) and centrifuged at 20°C, 500 g for 30 min. The PBMC interfaces were washed twice with PBS and the pellets were re-suspended in PBS. Aliquots were counted and dry pellets (2 × 10^5 ^cells) were stored under liquid nitrogen.

### RNA preparation

Total RNA was isolated from PBMC pellets following the detailed manufacturer's protocols in the Promega RNAgents Total Isolation System.

### Microarray Assay

RNA quality was confirmed using an Agilent RNA BioAnalyzer 2100. The target samples were prepared following GeneChip One-Cycle Target Labeling protocol (Affymetrix). All 15 samples were then hybridised to Affymetrix GeneChip HG-U133A and HG-U133B arrays. The arrays were washed and stained using Affymetrix protocols on the Fluidics Station 400 and scanned on the Gene Array Scanner 2500.

### Analysis: Analysis

GCOS1.1.1 software (Affymetrix) was used to generate raw data from scanned images. Data analysis was performed using FunAlyse, an in-house built automated pipeline in Sir Henry Wellcome Functional Genomics Facility (SHWFGF), University of Glasgow.

This analysis consists of the Robust Multichip Average (RMA) normalisation [16], followed by the identification of differentially expressed genes using the Rank Products (RP) method [17], performed for A and B chips separately. Briefly, the RP method sorts Affymetrix probe-sets by geometric mean of their ranks, calculated over all possible between-chip comparisons contributing to the disease vs. control comparison, where ranks are calculated after sorting probe-sets by log fold-change between each CFS and each control sample [17]. This method has been proven superior to others in situations where compared conditions are represented by small numbers of replicated samples [17-19]. Subsequently, the RP expression profiles for chips A and B chips were merged and trimmed at the significance cut-off of false discovery rate, FDR < 0.01 and fold change, FC > 1.5. The fold-change is initially calculated as an antilog of a mean log fold-change over all possible between-chip comparisons contributing to the disease vs. control comparison after RMA normalisation. It is then corrected using a procedure to compensate for RMA specific distortion of fold-change values [20].

The above RP-generated expression profile was then analysed with the Ingenuity Pathway Analysis (IPA) software (Ingenuity^® ^Systems, ) in order to identify possible biological processes associated with differentially expressed genes. The initial set of differentially expressed, redundant Affymetrix probe-sets was first mapped to Entrez Gene identifiers [21] and then submitted to the IPA server. Here, each Entrez Gene identifier was mapped to its corresponding object in the Ingenuity Pathways Knowledge Base (IPKB), which resulted in conversion of the initial expression profile into a shorter dataset of well-characterised, non-redundant "focus" genes. These genes were then overlaid onto a global molecular network developed from information contained in the IPKB and a number of small networks (up to 35 genes in total) were then algorithmically generated, based on their connectivity. Subsequently, for each network the genes associated with biological functions were identified and Fisher's exact test was then used to calculate p-values, determining the probability that each biological function assigned to a given network is due to chance alone. The whole dataset of the "focus genes" was then analysed in order to identify its most representative gene functional classes, as well as the most representative canonical pathways. The "focus" genes associated with biological functions were identified and the Fisher's exact test was then used to calculate p-values determining the probability that each biological function assigned to the "focus" gene data-set is due to chance alone. Similarly, the "focus" genes associated with canonical pathways were identified but the significance of that association was measured in two ways, (i) A ratio of the number of genes from the dataset that map to a given pathway, divided by the total number of genes that map to that pathway, is displayed, and (ii) the Fisher's exact test was used to calculate p-values determining the probability that the association between the genes in the dataset and a given canonical pathway is explained by chance alone. For all the above IPA analyses the Fisher exact test p-values were converted to the score equal to -log(p-value).

The data discussed in this publication have been deposited in NCBI's Gene Expression Omnibus [22] and are accessible through GEO Series accession number GSE14577 .

### Validation of defensin-α1

An additional cohort of 10 patients with CFS and 10 age- and sex-matched controls were recruited for Reverse Transcription-Polymerase Chain Reaction (RT-PCR) and western blot assays, in order to verify the microarray data for one of the putative biomarkers, defensin-α1.

### RT-PCR

cDNA synthesis and PCR were performed as described previously [23,24] and primers generated an amplified PCR product of the expected size 288 bp. The defensin-α1 oligonucleotide primers used were:

sense 5'-CAAGAGCTGATGAGGTTGCT-3'

antisense 5'-GAAGGTACAGGAGTAATAGC-3'.

### Western Blot

PBMC pellets were re-suspended in sample-reducing buffer (1 ml glycerol, 0.5 ml β-mercaptoethanol, 3 ml 10% SDS, 1.25 ml 1 M Tris-HCl, pH 6.7) and boiled for 5 min. The cell lysates were loaded onto 10% PAGE gels, each track equivalent to 2 × 10^5 ^PBMC. PAGE gels were assessed for equal protein loading by coomassie blue staining. The gels were then electrophoretically transferred onto PVDF membranes (BioRad), for 2 hours. Blots were blocked with 10% normal goat serum for 30 min, and then incubated for 2 hours at room temperature with a monoclonal antibody to human neutrophil defensin α1–3 (1:100, clone D21, HyCult Biotechnology, Netherlands), diluted in TBS/0.05% Tween-20. Proteins were detected with a 1:1000 dilution of alkaline phosphatase-conjugated goat anti-mouse IgG (Jackson Immunoresearch Laboratories, PA, USA) and protein bands were detected using SIGMA FAST BCIP/NBT.

## Results

### Identification of differentially expressed genes

A number of genes were significantly over- or under-expressed in CFS patients, as compared to healthy controls, in genome-wide microarray analysis. The Rank Products analysis revealed that 413 Affymetrix probe-sets were significantly up-regulated and 189 significantly down-regulated (602 in total) in the CFS samples. Of the significantly up-regulated probe-sets, 141 showed the expression changed at least 2-fold and 20 at least 3-fold. The corresponding numbers for the down regulated probe-sets are 57 and 2, respectively.

### IPA results

Annotating the above list of 602 differentially expressed probe-sets with EntrezGene IDs and subsequent submission to the IPA server resulted in successful mapping of the above probe-sets to 366 non-redundant "focus" genes with records in the IPA database. Of these, 286 genes were significantly up-regulated and 80 significantly down-regulated in the CFS samples. Of the significantly up-regulated genes, 105 genes showed that expression changed at least 2-fold and 16 genes at least 3-fold. The corresponding numbers for the down-regulated genes are 25 and 3, respectively. The ten most highly up-regulated genes and the three most highly down-regulated genes are presented in Table 1. The complete list of 366 genes (together with the raw data) is accessible through GEO Series accession number GSE14577.

**Table 1:** Top 10 up-regulated genes (+) and top 3 down-regulated genes (-) in post-infectious CFS patients

**Gene**	**FC**	**NCBI Accession Number**
***Top 10 up-regulated genes***		
Lactotransferrin (LTF)	+7.2	NM_002343
Defensin α1 (DEFA1)	+6.0	NM_004084
Charcot-Leyden Crystal protein (CLC)	+5.6	NM_001828
Haemaglobin, gamma A (HBG1)	+4.8	NM_000559
CEACAM8	+4.1	NM_001816
Integrin α2B (ITGA2B)	+4.1	NM_000419
Haemaglobin, gamma G (HBG2)	+3.9	NM_000184
Defensin α4 (DEFA4), corticostatin	+3.9	NM_001925
Integrin β3 (ITGB3), CD61	+3.8	NM_000212
Chemokine (C-X-C) receptor 4 (CXCR4)	+3.6	NM_001008540
***Top 3 down-regulated genes***		
RPS26	-4.2	NM_001029
ZNF294	-3.8	AK023499
HLA-DQA1	-3.5	NM_022122

**Figure 1:**
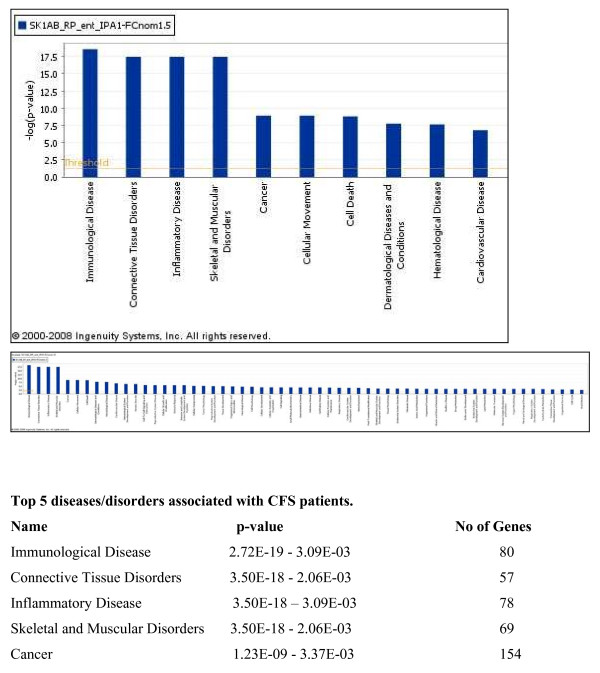
**High-level functional categories involved in this IPA analysis**. Y-axis displays -(log) significance. A) Top 10 biofunctions for CFS patients and B) total biofunctions for CFS patients. Taller bars are more significant than shorter bars. Functions are listed from most significant to least and the horizontal line denotes the cut-off for significance (p < 0.05).

**Figure 2:**
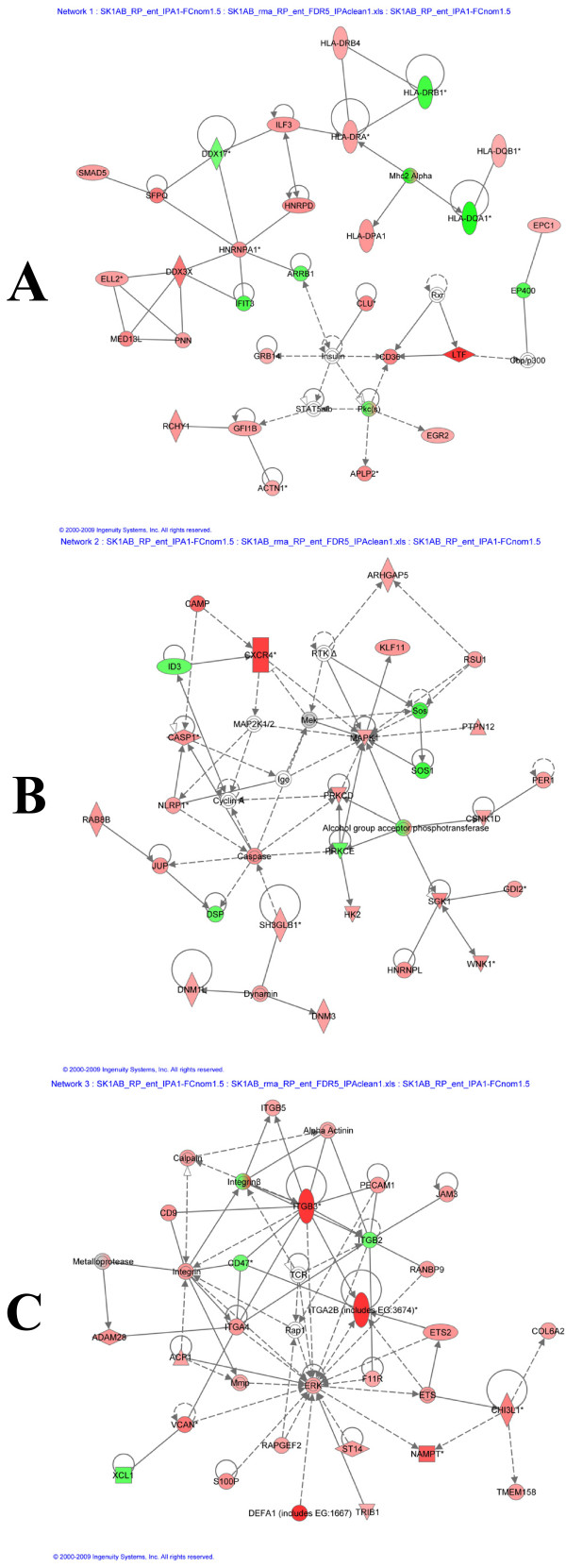
**Differentially expressed gene networks**. Networks showing differentially up-regulated genes (red) and down-regulated genes (green) in post-infectious CFS patients, whereby A) includes lactotransferrin (LTF, up-regulated 7.2-fold), B) includes CXCR4 (up-regulated 3.6-fold) and C) includes the integrins α2B and β3 (up-regulated 4.1 and 3.8-fold, respectively) and defensin-α1 (up-regulated 6-fold).

To investigate the biological context of genes differentially expressed in CFS patients, the IPA software was used, as described in Methods. Figure 1 presents functional classes associated with CFS patients. The network analysis revealed the existence of 21 networks with the significant score between 9 and 47, which together contain 349 out of the 366 genes. The top three networks, containing 5 of the top 10 up-regulated genes are presented in Figure 2 and include lactotransferrin (up-regulated 7.2-fold), CXCR4 (up-regulated 3.6-fold), the integrins α2B and β3 (up-regulated 4.1 and 3.8-fold, respectively) and defensin-α1 (up-regulated 6-fold).

### Altered bio-systems in CFS

The gene signature of CFS highlighted changes in gene expression in three main areas: oxidative stress, apoptosis and viral-like immune dysfunction. These genes are summarised in Table 2.

**Table 2:** Three altered bio-systems in CFS

**Oxidative Stress Genes**
***Up-regulated***
Prostaglandin Synthase: COX-1 and 2, haemoglobin gamma A and gamma G
***Down-regulated***
Glutathion-s-transferase

**Apoptosis Genes: *Up-regulated***

Annexin -A3, -A5, Serine/threonine kinase 17b, Histones 1&2, Protein S (alpha), Serum deprivation response (Phosphatidylserine binding protein) Caspase 1, TGFβ1 Death effector filament forming CED4-like apoptosis protein, Complement 3a receptor 1, Early growth response 1, TNF-A1P3, -RSF17, -SF4

**Immune Dysfunction Genes **(including markers of viral immuno-modulation/evasion)

***Up-regulated***
DAF (CD55) & CD46
Antigen processing via MHC class II (MHC II DP α1 and DR α)
IL-12, IL-13 & IL-6 biosynthesis
***Down-regulated***: The MHC-1 system including:
Natural Killer cell receptors (KIR)
TCR complex (T-cell receptors α, β, δ, γ)
NO production (up-regulation of arginase I & II)
Flavohaemoprotein B5 Leukocyte-derived arginine aminopeptidase (L-rap)

### Oxidative stress

There is a large body of evidence from researchers in CFS indicating that oxidative stress contributes to disease progression and resultant symptoms [25-29]. Our gene array confirms evidence of oxidative stress (Table 2) with a large change in the genes for prostaglandin-endoperoxide synthase 1 and 2 (also known as COX-1 and -2). These enzymes are responsible for the regulation of synthesis of the pro-inflammatory mediators, prostaglandins. The inducible form of COX-2 catalyses the conversion of arachidonic acid to prostaglandins, the release of prostanoids, which sensitise peripheral nociceptor terminals and produce localised pain hypersensitivity. COX-2 has also been shown to be over-expressed at sites of inflammation and in various tumours, with increased expression of the major arachidonate metabolite, prostaglandin E2 (PGE2). COX-2 derived PGE2 is also known to increase expression of decay-accelerating factor (DAF or CD55). DAF is one of the key protective complement receptor genes and an immune regulator, which is of interest as DAF was up-regulated on our B chip. This is highly significant as regards immune suppression/subversion and cell dissemination, which is discussed further in the section on immune-modulation in CFS below. Furthermore, a recent study by Maes *et al. *[8] showed that TNFα-stimulated lymphocytes cultured from CFS patients resulted in over-expression of NFkappaB (NFκB). COX-2 is responsible for multiple inflammatory actions, whilst NFκB is the major upstream mechanism which regulates inflammatory and oxidative stress mediators, such as pro-inflammatory cytokines, and COX-2 and inducible NOS (iNOS) production. The over-expression of genes for oxidative stress in CFS is also consistent with the up-regulation of the haemoglobin genes, especially gamma haemoglobin, which is usually only expressed in the foetus. Additional up-regulated genes involved in oxidative stress were Leukotriene B4, cytochrome P450, superoxide dysmutase and the mono-oxygenases. Indirect evidence of SO2- attack comes from the up-regulation of gamma, and alpha haemoglobin, especially gamma haemoglobin. This protein's unique property of increased oxygen binding could be induced as a result of oxidative attack.

### Apoptosis

There is active apoptosis in the cells of patients with CFS and consistent with this is the finding that many apoptosis-associated genes are up-regulated, including Caspase 1 (Table 2). Several factors may trigger apoptosis: cellular damage, infection with a virus and homeostasis. The signal for apoptosis can be from within the cell, from surrounding tissue or from a cell belonging to the immune system. Among the top 100 up-regulated genes there is a predominance of apoptosis-associated genes. Apoptotic cells and their nuclei shrink and often fragment. They can then be efficiently phagocytosed by macrophages or neighbouring cells. From the gene array there is also evidence of macrophage activation, with the up-regulation of Toll-like receptor (TLR)-4 and TLR-8, and monocyte-to-macrophage differentiation genes. As to exactly which cells are undergoing apoptosis, theories abound. Kennedy and co-workers have previously reported increases in neutrophil apoptosis in CFS [30]. It may be that it is these groups of cells which are the targets of apoptosis, and our array data certainly includes many genes related to neutrophils, as well as eosinophils.

### Immune-modulation

Immune evasion is a well-documented mechanism by which viruses, bacteria, parasites and cancer cells avoid destruction by the host. Immune modulations can be varied and complex. They include targeted disruption of T-cell function, suppression of T-cell cytotoxic death receptors, induction of apoptosis proteins, inhibition of innate immune systems such as dendritic cell and natural killer (NK) receptors. From this study, several genes associated with the immune system are shown to be up-regulated in patients with CFS, thus indicating possible functional alterations (Table 2). Among those which have been identified are 2 genes belonging to the Major Histocompatibility Complex (MHC); HLA-DRB4 and HLA-DQB1 and these genes encode β-chains on MHC class II molecules. MHC class II molecules are found on antigen-presenting cells, namely B-cells, dendritic cells and macrophages. Antigens associated with MHC class II are presented to CD4+ cells, a subset of T-cells. Previous studies have identified perturbations in the expression of MHC class II genes in patients with CFS. Steinau and co-workers indicated a down-regulation of MHC class II molecules, however this work did not identify individual MHC class II genes [31]. Subsequently, Smith *et al. *[32] indicated increased expression of HLA-DQB1 and have also highlighted a number of other MHC genes altered in CFS. The data presented in this current study therefore support some of these findings, but also identify changes in the expression of HLA-DRB4 in patients with CFS, which has previously not been shown.

In addition to the MHC, within the top up-regulated genes there are several genes encoding immune proteins which belong to the innate immune system. Patients with CFS have been suspected to have subtle immune dysfunction. Altered NK cell function in CFS patients has been reported previously by Morrison *et al*. [33], and is verified by the significant down-regulation of NK KIR receptors in our gene array (Table 2). This data helps to confirm that immunomodulatory gene expression is significantly different in CFS patients, as compared to healthy individuals. Immune dysfunction appears to be present in patients with CFS in the absence of any particular pathogen. The classical Th1 to Th2 switch allows intracellular pathogens to evade detection. As mentioned above, DAF is a regulator of the complement system and is an immune regulator. DAF appears to inhibit NK cells [34] and is known to be one of the most potent activators of tumourigenesis/viral dissemination. DAF is also known as a receptor for certain viruses (including enteroviruses) and other microorganisms [35-37].

The immune modulation in post-Lyme disease fatigue is remarkably similar to the gene signature presented here for patients with CFS [38]. It is known that gene expression profiles of total mixed PBMC populations may mask gene expression in subsets of cells [39]. This study, however, demonstrates very high expression of a number of MHC class II genes normally observed in the minor proportions of the PBMC, indicating significant changes within these cell groups.

Up-regulated immune-related genes in patients include lactotransferrin, defensin-α1 and integrins. Lactotransferrin is an iron-binding protein responsible for immunological reactions, while defensins are a family of microbicidal and cytotoxic peptides released in response to viral and bacterial infections. Defensins are also involved in the anti-microbial defence of epithelial surfaces (i.e. respiratory and urinogenital tracts). Patients with CFS are known to have increased susceptibility for infections and candidiasis is frequently reported [40].

Integrins are extracellular matrix proteins involved in cell adhesion and also activate cytosolic signal cascades for cell growth and regulation. Arginase is a key enzyme considered to be active in immune modulation and is also involved in NK cell function. Other notable up-regulated genes include haemoglobins, especially foetal haemoglobin, and this may suggest a response to increased oxidative stress. Another family of up-regulated genes relate to cellular apoptosis and serine-threonine kinase is a member of brain kinase family of protein implicated in neural apoptosis and formation of corpora amylacea. Of the down-regulated genes, ribosomal protein gene and zinc finger protein genes are involved in cell cycle and cellular growth.

### Validation of gene array data using RT-PCR and western blotting

**Figure 3:**
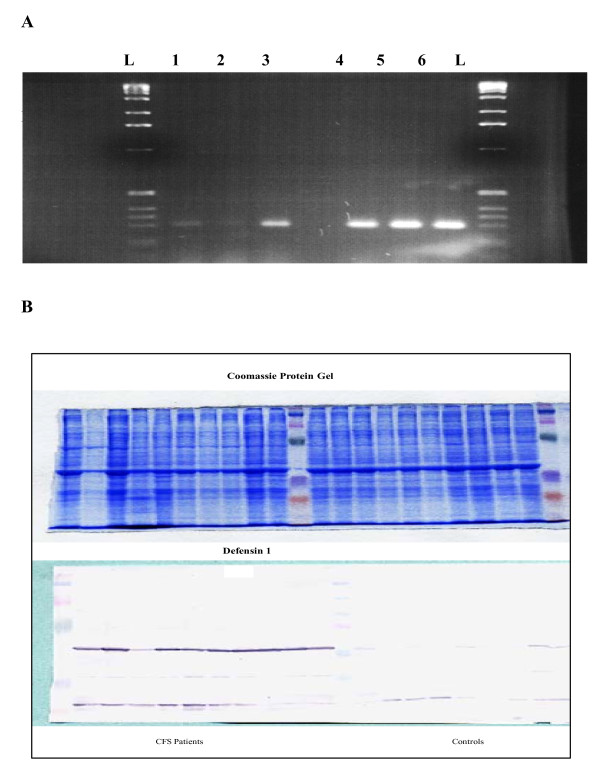
**RT-PCR assay and western blot analysis**. Detection of over-expression of defensin-α1 in CFS patients compared to normal healthy controls. A) 25 μl of each PCR product was electrophoresed through a 2.5% agarose gel, ethidium bromide stained and UV visualised. Lanes: L: DNA size ladder; 1–3: Healthy controls; 4–6: Patients with CFS. B) Western blot analysis. Each lane contained 2 × 10^5 ^PBMC and equivalent protein loading of all samples was confirmed by coomassie blue staining. Left-hand side of blot: 10 CFS patients; right-hand side of blot: 10 healthy controls.

Two members of the defensin gene family were found to be amongst the most highly over-expressed genes in the microarray; defensin-α1 and -α4. Defensins are a family of cytotoxic peptides found in the microbicidal granules of neutrophils and macrophages, and are released in response to viral and bacterial infections. These proteins are thus likely to play a role in phagocyte-mediated host defence. As a result of the high defensin levels identified in the microarray data, defensin-α1 was chosen for further analysis. We aimed to confirm this data at the RNA and protein level, using another cohort of blood samples from 10 CFS patients and 10 normal healthy controls. Oligonucleotides and antibodies specific to defensin-α1 were used to detect and compare changes at the RNA and protein level using RT-PCR and western blotting, respectively. Figure 3A demonstrates up-regulation of defensin-α1 at the RNA level in patients with CFS, when compared to normal healthy controls. Moreover, the immunoblot in Figure 3B demonstrates up-regulation of defensin-α1 protein in all CFS patients (except for one), when compared to controls that showed little or no defensin protein. Equal loading was confirmed using coomassie blue staining.

## Discussion

A comprehensive gene signature of non-psychiatric patients with CFS has been generated by a whole-genome microarray assay. The microarray data presented here illustrates differences in gene expression across the human genome between post-infectious, non-psychiatric male patients with CFS and healthy controls. Analysis of the gene signature of CFS suggests that three significant pathways are altered in patients: oxidative stress, apoptosis and viral-like immune modulation. Previously, Gu *et al. *[41] performed a 588-gene microarray whereby patients with spondyloarthropathy, rheumatoid arthritis and psoriatic arthritis were compared to normal healthy subjects. Of interest was their finding that expression of CXCR4 was unexpectedly high among all arthritis subjects. This gene encodes a CXC chemokine receptor that is specific for one ligand (stromal cell-derived factor-1), and CXCR4 is known to act with CD4 protein to support HIV entry into cells. DNA microarray studies by Watanabe and co-workers [42] demonstrated significant up-regulation of the CXCR6 motif in ulcerative colitis patients, whilst other work has indicated that the CXCL12/CXCR4 interaction is involved in several inflammatory conditions, including inflammatory bowel disease [43]. As a result, the finding from our study (that CXCR4 was significantly up-regulated in male CFS patients) indicates that this gene may not be specific to CFS. It is noted, however, that in the CFS studies by Kerr *et al. *(44, 45), CXCR4 was also up-regulated.

The overall picture emerging from these results point to a significant, albeit phenotypically subtle, perturbation of function in relation to microbial defence, viral immuno-surveillance and cell growth amongst patients with post-infectious chronic fatigue. A possible criticism of the work is that female patients were excluded. As already explained, the primary reason for exclusion was to avoid any confounding endocrine influence on gene signature. Previous research has found few gender-related differences in terms of symptom severity. There was no gender effect in a prospective cohort study of patients with post-infectious fatigue attending primary care [44]. In general, demographic, clinical and psychosocial factors do not distinguish men from women patients with CFS [45] and we believe that the results of our research apply to CFS patients of both genders.

## Conclusion

Recent studies have attempted to determine the molecular basis of CFS [46-49]. Table 3 illustrates overlapping genes observed in CFS patients in our analysis and in previous microarray studies (and one filter array study), by other researchers. Although there is some overlap, overall there is a lack of correlation between different publications on gene arrays to date. Whether this reflects different patient populations and selection criteria remains unclear. It must be borne in mind, however, that many mRNAs have very short half-lives (i.e. less than 2 mins) and therefore what is observed in the microarray is a snapshot in time and may not necessarily reflect the chronic biological process inherent of this condition. It is necessary, therefore, to validate the microarray data by more established technologies such as RT-PCR, western blotting and ELISA, and use wider populations of carefully selected patients to validate the results.

**Table 3:** Commonly overexpressed CFS genes between current study and previous microarray studies

**Gow *et al. *(2009)**	**Vernon *et al. *(2002)**	**Kaushik *et al. *(2005)**	**Fang *et al. *(2006)**	**Kerr *et al. *(2008)**
**Dynactin1**	Dynactin1			
**ARF1**	ARF1			
**CEACAM family**		CEACAM family		
**Defensin family**			Defensin family	
**CXCR4**				CXCR4
**EGR1**				EGR1
**PRKAR1A**				PRKAR1A

The data presented here clearly illustrate important differences in gene expression between patients with post-infectious CFS and healthy controls. While these differences in gene signatures may offer a rational explanation for the symptoms, and confirm that CFS is a true and biological illness, gene microarray results do not imply that CFS is primarily a genetic disorder. In addition, the research data do not prove if differentially expressed genes are the predisposing cause or downstream effect of CFS. We believe, however, that the data presented here is an important first step forward towards the goal of identifying putative biomarkers to support the clinical diagnosis of CFS. Furthermore, the biological pathways identified by the over-expressed genes offer a rational understanding and possible explanation of the complex and often multi-systemic nature of CFS, which is common and remains a potentially disabling illness in search of an effective cure.

## List of Abbreviations

CFS: Chronic Fatigue Syndrome; DAF: Decay Acceleration Factor; FC: Fold Change; FDR: False Discovery Rate; GMN: Global Molecular Network; IPA: Ingenuity Pathway Analysis; MHC: Major Histocompatibility complex; NFκB: Nuclear Factor kappa B; PBMC: Peripheral Blood Mononuclear Cells; RT-PCR: Reverse Transcription-Polymerase Chain Reaction; RMA: Robust Multichip Analysis; TLR: Toll-Like Receptor.

## Competing interests

The Court of the University of Glasgow has filed a patent entitled "Materials and Methods for Diagnosis and Treatment of Chronic Fatigue Syndrome" Patent File Number GB0502042.5.

## Authors' contributions

AC, CC and JG participated in the design and coordination of the study, carried out the molecular genetic studies, participated in the sequence alignment and helped to draft the manuscript. CC carried out the immunoassays. PH carried out the IPA analysis and performed the statistical analysis. SH carried out the analysis and interpretation of IPA analysis and drafted the manuscript. PB participated in the design of the study and drafted the manuscript. All authors read and approved the final manuscript.

## Pre-publication history

The pre-publication history for this paper can be accessed here:


